# AI-powered insights in pediatric nephrology: current applications and future opportunities

**DOI:** 10.1007/s00467-025-06911-1

**Published:** 2025-09-16

**Authors:** Arwa Nada, Yamen Ahmed, Jieji Hu, Darcy Weidemann, Gregory H. Gorman, Eva Glenn Lecea, Ibrahim A. Sandokji, Stephen Cha, Stella Shin, Salar Bani-Hani, Sai Sudha Mannemuddhu, Rebecca L. Ruebner, Aadil Kakajiwala, Rupesh Raina, Roshan George, Rim Elchaki, Michael L. Moritz

**Affiliations:** 1 Department of Pediatrics, Division of Pediatric Nephrology, Loma Linda University Children’s Hospital (LLUCH), Loma Linda University (LLU), 11175 Campus St. A1120H, Loma Linda, CA 92350 USA; 2 Case Western Reserve University, Cleveland, OH USA; 3 College of Medicine, Northeast Ohio Medical University, Rootstown, OH USA; 4 Department of Pediatrics, Children’s Mercy Kansas City, and University of Missouri-Kansas City School of Medicine, Kansas City, MO USA; 5 Office of the Surgeon General, U.S. Army, Falls Church, USA; 6 Department of Pediatrics, Division of Pediatric Nephrology, UCSF Benioff Children’s Hospital, San Francisco, CA USA; 7 Department of Pediatrics, Taibah University College of Medicine, Medinah, Saudi Arabia; 8 Department of Pediatrics, Division of Pediatric Nephrology, Penn State Health Children’s Hospital, Penn State, Hershey, PA USA; 9 Department of Pediatrics, Division of Nephrology, Emory University and Children’s Healthcare of Atlanta, Atlanta, GA USA; 10 Department of Pediatrics, Division of Pediatric Nephrology, Loma Linda University Children’s Hopistal, Loma Linda University, Loma Linda, CA USA; 11 Department of Medicine, East Tennessee Children’s Hospital, University of Tennessee, Knoxville, TN USA; 12 Department of Pediatrics, Division of Nephrology, Johns Hopkins University School of Medicine, Baltimore, USA; 13 Department Pediatrics, Division of Critical Care Medicine, Children’s National Hospital, Washington, D.C USA; 14 Department of Pediatrics, Division of Pediatric Nephrology, Akron Children’s Hospital, Akron, OH USA; 15 Department of Pediatrics, Division of Nephrology, Akron Children’s Hospital, Akron, OH USA

**Keywords:** Artificial intelligence, Kidney injury, Pediatrics, Nephrology

## Abstract

Artificial intelligence (AI) is rapidly emerging as a transformative force in pediatric nephrology, enabling improvements in diagnostic accuracy, therapeutic precision, and operational workflows. By integrating diverse datasets—including patient histories, genomics, imaging, and longitudinal clinical records—AI-driven tools can detect subtle kidney anomalies, predict acute kidney injury, and forecast disease progression. Deep learning models, for instance, have demonstrated the potential to enhance ultrasound interpretations, refine kidney biopsy assessments, and streamline pathology evaluations. Coupled with robust decision support systems, these innovations also optimize medication dosing and dialysis regimens, ultimately improving patient outcomes. AI-powered chatbots hold promise for improving patient engagement and adherence, while AI-assisted documentation solutions offer relief from administrative burdens, mitigating physician burnout. However, ethical and practical challenges remain. Healthcare professionals must receive adequate training to harness AI’s capabilities, ensuring that such technologies bolster rather than erode the vital doctor-patient relationship. Safeguarding data privacy, minimizing algorithmic bias, and establishing standardized regulatory frameworks are critical for safe deployment. Beyond clinical care, AI can accelerate pediatric nephrology research by identifying biomarkers, enabling more precise patient recruitment, and uncovering novel therapeutic targets. As these tools evolve, interdisciplinary collaborations and ongoing oversight will be key to integrating AI responsibly. Harnessing AI’s vast potential could revolutionize pediatric nephrology, championing a future of individualized, proactive, and empathetic care for children with kidney diseases. Through strategic collaboration and transparent development, these advanced technologies promise to minimize disparities, foster innovation, and sustain compassionate patient-centered care, shaping a new horizon in pediatric nephrology research and practice.

**Graphical Abstract:**

**Figure:**
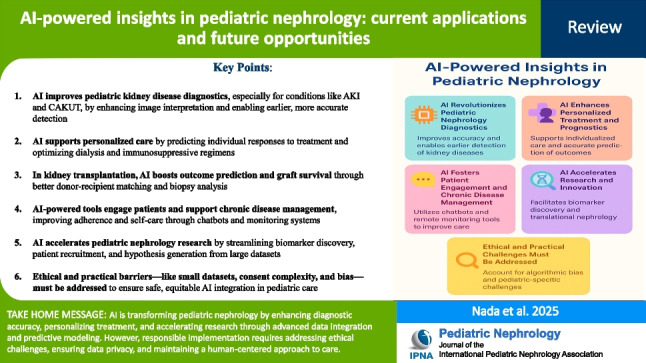
A higher resolution version of the Graphical abstract is available as Supplementary information

**Supplementary Information:**

The online version contains supplementary material available at 10.1007/s00467-025-06911-1.

## Artificial intelligence: the future of pediatric nephrology

In the ever-evolving field of medicine, pediatric nephrology stands at a promising crossroads, brought about by the advent of artificial intelligence (AI). As AI continues to reshape various medical specialties, its integration into pediatric nephrology is proving to be not just beneficial but transformative. The promise of AI in enhancing diagnostic accuracy, personalizing treatment plans, and improving operational efficiencies cannot be overstated. However, as we venture down this path, it is crucial to consider both the immense potential and the challenges that lie ahead.

**Fig. 1:**
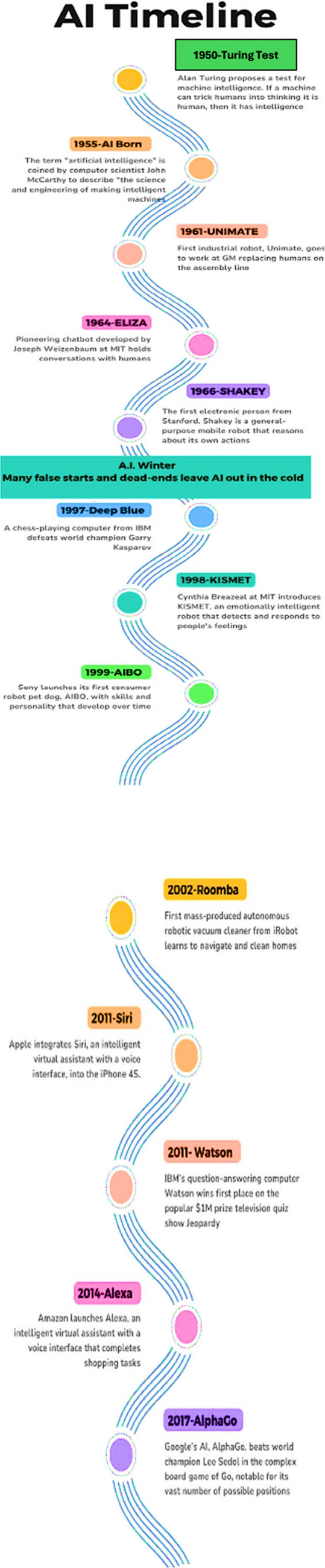
AI timeline

AI is not a new concept; its roots trace back to the 1950s when Alan Turing introduced the idea of machines simulating human intelligence through his famous “Turing Test.” This test was designed to assess whether a machine could exhibit behavior indistinguishable from that of a human. Since then, AI has evolved dramatically, with advancements in computing power and data science propelling its development. While early AI concepts focused on logic and problem-solving, today’s AI has expanded into numerous fields, including healthcare, offering transformative opportunities like never before. Figure 1 shows the AI timeline.

## Understanding AI: definitions and types

**Fig. 2:**
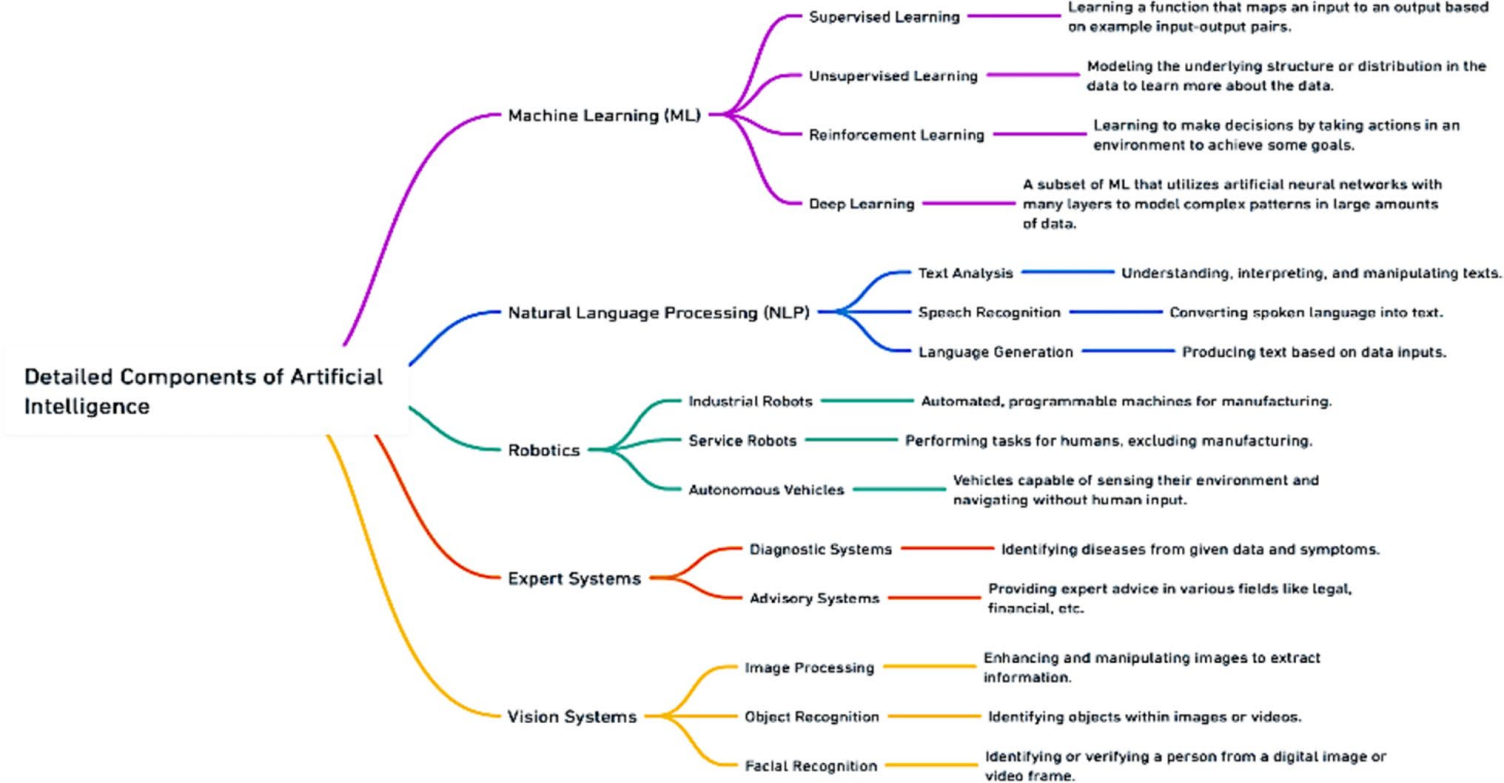
Basic concepts and classifications of AI

Before delving deeper into AI’s applications, it is essential to understand what AI encompasses and the various forms it takes. AI broadly refers to the capability of a machine to imitate intelligent human behavior [1]. It is designed to perform tasks that typically require human intelligence such as understanding language, recognizing patterns, solving problems, and learning from experience (Fig. 2).

*Machine learning* (ML), a subset of AI, involves algorithms that allow software applications to become more accurate in predicting outcomes without being explicitly programmed. ML uses statistical techniques to give computers the ability to “learn” from data. ML includes 2 main categories: supervised and unsupervised learning (Table 1 ).

**Table 1:**
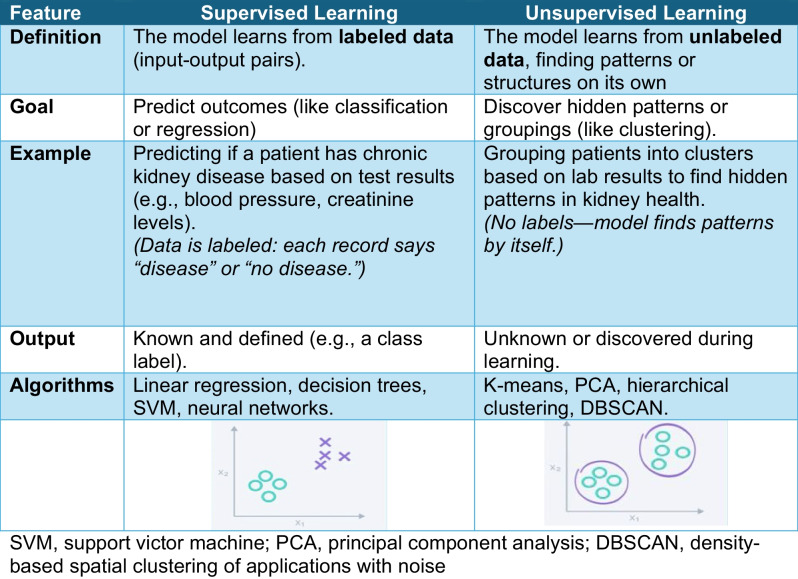
Comparison between supervised learning and unsupervised learning

*Deep learning* (DL), a specialized form of machine learning, utilizes neural networks with three or more layers. These neural networks attempt to simulate human decision-making processes. Deep learning is particularly effective in identifying patterns in unstructured data like images and sounds. Table 2 shows the differences between ML and DL.

**Table 2:** Comparison between machine learning and deep learning

Aspect	Machine learning (ML)	Deep learning (DL)
**Definition**	A subset of AI that involves statistical methods to enable machines to improve with experience	A subset of machine learning that uses neural networks with many layers to learn data representations
**Approach**	Uses algorithms to parse data, learn from it, and make decisions	Employs deep neural networks to learn data representations
**Data handling**	Effective with small to medium-sized datasets	Requires large datasets to perform well
**Feature engineering**	Requires manual feature extraction and selection	Automates feature extraction and representation
**Computational resources**	Generally, requires less computational power	Needs high computational power and specialized hardware like GPUs
**Interpretability**	Models are often easier to interpret	Models can be complex and act like “black boxes”
**Applications**	Spam filtering, recommendation systems, fraud detection	Image recognition, voice recognition, natural language processing
**Outcome measures and applications**	**Predictive accuracy:** traditional ML models can produce robust predictions when the data is well-structured and the number of relevant features is not too large. **Interpretability:** many ML algorithms offer clearer insight into which variables drive the model’s predictions, which can be important for clinical acceptance and regulatory considerations. **Targeted applications:** often used for risk stratification, early detection of complications, and identifying subgroups of patients who respond differently to treatments	**High-dimensional data performance:** DL models shine in settings where large amounts of complex data—like imaging or continuous monitoring data—are available. **Automated feature extraction:** because DL can learn intricate patterns without manual feature selection, it may identify previously unrecognized predictors of disease progression. **Greater data requirements:** deep neural networks typically need larger datasets to avoid overfitting and to achieve reliable performance. When used appropriately, DL can achieve higher accuracy in specific tasks (e.g., image-based diagnostics), but the trade-off is reduced interpretability compared to many traditional ML methods
**Examples in pediatric nephrology**	Could employ a random forest model to predict which children with chronic kidney disease (CKD) are most at risk of rapid progression based on discrete clinical and laboratory data. This approach might highlight specific risk factors (e.g., proteinuria levels and blood pressure readings) that can guide individualized treatment plans	A convolutional neural network might analyze renal ultrasound images to detect early structural abnormalities or segment kidney volumes, potentially revealing subtle changes that clinicians or simpler algorithms might miss. While potentially more accurate, such a model may be less transparent about how it arrives at its conclusions
**Types**	Supervised, unsupervised, semi-supervised, reinforcement learning	Convolutional neural networks (CNNs), recurrent neural networks (RNNs), generative adversarial networks (GANs)

*Natural language processing* (NLP) enables machines to understand and interpret human language. In medical applications, NLP is used to extract meaningful information from clinical texts such as patient records and research papers.

*Robotics* involves programming robots to perform tasks that are typically done by humans. In healthcare, robotics is used in surgeries, therapy, and handling hazardous materials, enhancing precision and safety.

*Computer vision* is the AI field that trains computers to interpret and understand the visual world. In medical imaging, computer vision helps to analyze images more accurately and faster than human radiologists.

The integration of these AI components in medicine not only enhances the efficiency and effectiveness of healthcare services but also opens new avenues for personalized medicine, where treatments and interventions are tailored to individual patient needs. The potential of AI in revolutionizing healthcare is vast, with ongoing research and development aimed at further improving patient care, reducing healthcare costs, and making healthcare systems more resilient and responsive to emerging challenges.

## Transforming diagnostics

AI’s role in revolutionizing diagnostics is particularly notable in pediatric nephrology. With sophisticated image analysis and pattern recognition capabilities, AI algorithms can detect minute changes in kidney morphology—changes so subtle they might elude even the most trained eyes. These capabilities may allow earlier detection of kidney anomalies and diseases beyond current capabilities. Beyond diagnostics, AI’s predictive analytics are tailoring treatments to the individual needs of pediatric patients. By analyzing vast datasets of patient history, genetics, and treatment outcomes, AI models may predict disease progression and optimize treatment plans [2–4]. This not only enhances the efficacy of interventions but also minimizes potential side effects, offering a level of personalization that was previously unattainable.

AI has demonstrated significant potential in diagnosing various pediatric nephrology conditions through its ability to process complex clinical, imaging, and laboratory data. For example, acute kidney injury (AKI) can be challenging to detect early due to nonspecific clinical signs and the lagging of serum creatinine behind the kidney insult. ML algorithms trained on large datasets of electronic health records (EHRs) can predict AKI onset by analyzing real-time patient data, such as creatinine trends, urine output, and hemodynamic parameters. A 2019 study by Tomašev et al. developed an AI model using deep learning technology that predicted more than 50% of AKI episodes up to 48 h before clinical recognition and predicted 90.2% of AKI episodes requiring dialysis [5]. Another study by Tsai et al. demonstrated the use of AI models for early detection and screening of pediatric kidney diseases, highlighting the utility of AI in recognizing pathologies from ultrasound images effectively [6]. Similarly, Kuo et al. [7] discussed AI’s role in predicting kidney function and disease classification through ultrasound imaging, suggesting that AI can significantly aid in risk prediction and management of chronic kidney diseases (CKD) in pediatric patients.

Another notable application is in diagnosing congenital anomalies of the kidney and urinary tract (CAKUT), the leading cause of CKD in children. AI-powered image analysis tools use DL models to interpret ultrasound and MRI scans, identifying structural abnormalities such as hydronephrosis, kidney dysplasia, and vesicoureteral reflux. These models can assist radiologists by reducing interpretation variability and increasing diagnostic accuracy. DL has emerged as a powerful tool for detecting and characterizing pediatric kidney abnormalities using ultrasound. For example, Miguel et al. [8] showed how deep neural networks can accurately predict fetal kidney anomalies from a relatively small prenatal ultrasound dataset, achieving an AUC over 91% by employing multi-class interpretation and explainable AI methods. Moreover, Song et al. [9] demonstrated that convolutional neural networks (CNN) can effectively segment kidney and hydronephrosis regions in ultrasound images, enabling automated calculation of the “hydronephrosis area to kidney parenchyma ratio (HARP),” thus facilitating more reproducible assessments of hydronephrosis severity. A range of other studies emphasize the utility of multi-instance DL, which treats a patient’s multiple ultrasound slices (e.g., sagittal and transverse) as sets of images rather than relying on a single, potentially unrepresentative view. Yin et al. [10, 11] used this strategy to distinguish patients with congenital abnormalities of CAKUT from controls, achieving higher accuracy than single-view models and identifying clinically meaningful image regions via classification activation mapping. Moreover, a study by Kwong et al. applied ML to predict key clinical outcomes in patients with posterior urethral valves (PUV) using data from 2000–2020. Among 103 patients (median follow-up: 5.7 years), outcomes included CKD progression (25%), kidney replacement therapy (KRT) initiation (17%), and clean-intermittent catheterization (CIC) need (31%). ML models using early kidney and anatomical data outperformed traditional Cox regression, with high predictive accuracy for KRT (c-index = 0.95) and good performance for CKD progression (0.77) and CIC (0.70). External validation confirmed these results. A user-friendly online tool was developed for clinical use. The study supports the feasibility of ML-driven clinical decision tools in PUV care [12]. Weaver et al. evaluated whether deep learning-derived features from postnatal kidney ultrasounds could improve prediction of CKD progression in boys with PUV. In a retrospective cohort of 225 patients (1990–2021), models using imaging features, clinical data, and a combination of both were developed. While clinical models—especially those using nadir creatinine—performed best, combining imaging and clinical data in an ensemble model further improved predictive accuracy (*C*-index = 0.82 at 6 months). The findings suggest that deep learning applied to ultrasound images can enhance early risk stratification for CKD progression in children with PUV [13].

Furthermore, adopting graph CNN and attention-based pooling can optimize instance-level feature extraction and improve diagnostic precision. Zhang et al. highlighted the strength of deep transfer learning, combined with conventional texture-based imaging features, to classify normal versus CAKUT-affected kidneys with an AUC exceeding 0.90 in some cases, indicating that hybrid approaches can enhance diagnostic performance even with moderate data sizes [14]. Collectively, these findings underscore the potential of deep learning to streamline prenatal and postnatal ultrasound evaluation, accurately detect a broad spectrum of pediatric kidney anomalies, support earlier interventions, and improve patient outcomes.

These AI systems not only accelerate the diagnostic process but also aim to reduce human error by providing second opinions. Advanced AI models are trained to detect subtle patterns that might be overlooked by human eyes, making them invaluable in clinical settings where quick and accurate diagnosis is critical. The incorporation of AI into pediatric kidney ultrasound practices promises not only to improve diagnostic accuracy but also to enhance patient outcomes by potentially enabling earlier intervention and tailored treatment plans.

## Kidney biopsy interpretation

AI is increasingly becoming a valuable tool in pediatric kidney biopsy and pathology, aiding in the accurate diagnosis and management of kidney diseases in children. AI technologies, especially in image analysis, are used to enhance the interpretation of biopsy samples, providing detailed insights that might be challenging for even experienced pathologists to detect. For instance, AI-assisted quantification and assessment of whole slide images can significantly improve the diagnosis of pediatric kidney diseases by providing precise measurements and identification of pathological features. A recent study by Feng et al. [15] introduced an AI method that assists pathologists in the assessment of pediatric kidney structures, which is crucial for diagnosing various kidney diseases accurately and promptly. This approach not only speeds up the diagnostic process but also enhances the accuracy of the diagnosis, which is vital for determining the appropriate treatment paths for pediatric patients. Moreover, De Bel et al. investigated the application of DL methods in classifying histopathological features of Wilms tumors using MRI data. Researchers trained convolutional neural networks on large datasets to detect malignancy and tissue differentiation patterns. Their model demonstrated high accuracy, significantly improving upon traditional diagnostic methods. The system supports radiologists by providing automatic, reproducible assessments. This approach enhances early detection and informs personalized treatment plans in pediatric nephrology [16]. Additionally, the recent advances in DL have greatly enhanced our ability to interpret kidney biopsy images, yielding promising applications in prognostication, transplant decision-making, and quantitative pathology. Testa et al. focused on IgA nephropathy, developing a neural network that autonomously extracted a histologic prognostic score for kidney failure from digitized biopsy images. By examining data from 442 patients over a median follow-up of nearly 7 years, they showed that the algorithm achieved an area under the ROC curve (AUC) of 0.84, which was noninferior to the well-established MEST-C scoring used in the International IgA Nephropathy Prediction Tool. Intriguingly, while the DL-derived score correlated well with tubulointerstitial damage (*r* = 0.41), it did not align with other pathologic features (e.g., mesangial hypercellularity, segmental sclerosis, and crescents) traditionally included in the MEST-C. The authors further noted that their model appeared to capture additional risk factors—particularly inflammation in fibrotic regions and hyaline casts—suggesting that AI may uncover new or underrecognized indicators of progression in IgA nephropathy [17].

In the setting of transplantation, Luo et al. leveraged whole-slide donor kidney biopsies to predict posttransplant kidney function for 219 deceased-donor recipients. Their model combined CNN (e.g., EfficientNet-B5) with donor/recipient clinical variables, yielding an AUC of 0.83 for stable estimated glomerular filtration rate (eGFR) prediction and 0.80 for predicting reduced graft function (RGF). Employing Grad-CAM revealed that deep learning algorithms emphasized tubular and interstitial regions near glomeruli, pointing to their significance in graft prognosis [18]. In parallel, Marsh et al. [19] targeted global glomerulosclerosis quantification—a key metric often assessed in donor kidney biopsies to decide whether to accept or discard an organ. After training on annotated images from 83 kidneys, their deep learning tool outperformed on-call pathologists in measuring percent glomerulosclerosis, reducing root mean square error by 14–22% and lowering the risk of erroneous organ discard by 37%. Collectively, these studies underscore the transformative potential of deep learning for refining both risk stratification and transplant allocation, highlighting AI’s growing role in elevating the accuracy and objectivity of kidney pathology.

The implementation of AI in this field not only helps in the standardization of pathological assessments but also reduces the variability and potential errors associated with human analysis. As AI technology continues to evolve, it holds the promise of further revolutionizing the field of nephropathology by enabling more sophisticated diagnostic capabilities and potentially even real-time pathological assessments during surgical procedures.

## Therapeutics

AI algorithms can analyze vast amounts of patient data, including genetic information, clinical history, and laboratory results, to create personalized treatment plans. This approach allows for more precise dosing and medication selection based on individual patient characteristics. Moreover, advances in AI can help increase patient engagement in their own care and improve compliance.

## Enhancing patient engagement

Chatbots hold considerable promise for transforming patient engagement and care delivery in medicine, as evidenced by multiple recent studies. By offering immediate, personalized support, these AI-driven tools can help patients with tasks like medication reminders, symptom monitoring, and answering routine health questions—all accessible through intuitive conversational interfaces. In particular, research shows chatbots can counter misinformation, improve communication with healthcare professionals [20], and deliver reliable healthcare information [21], though some studies caution that AI-generated references or specific clinical guidance may lack full accuracy [22]. Still, this technology’s accessibility and adaptability make it especially suitable for adolescent patients in fields like pediatric nephrology, where consistent therapy adherence is crucial yet often challenging. By engaging teens in real time with age-appropriate health tips and interactive educational modules, chatbots can reinforce treatment adherence and reduce the burden on overextended clinical teams [23]. Furthermore, adopting AI-based chatbots into chronic disease management programs, such as those for hypertension or kidney disease, has demonstrated improved self-management behaviors and patient satisfaction [24, 25]. As these studies collectively indicate, chatbots represent a powerful digital intervention capable of enhancing communication, empowering patient education, and ultimately supporting better outcomes in pediatric nephrology. Despite its promise, implementation of AI will require human clinical expertise and ethics for guidance before, during, and after its introduction to clinical practice.

## Predicting response to treatment

Ye et al. explored the development of machine learning models (MLM) to predict steroid-resistant nephrotic syndrome (SRNS) in 91 pediatric patients [26]. The best-performing model had 94% accuracy at predicting SRNS at the onset of nephrotic syndrome and used non-traditional albeit not widely available predictors. Whether such models would change practice on the waiting time for steroid response or the decision to biopsy remains an open question. Theoretically, a clinician armed with such knowledge could institute steroid-sparing interventions or perform a biopsy earlier.

Although Ye et al. focused on nephrotic syndrome, the principles they employed—aggregating multi-dimensional data (genomic, clinical, and lab results) and using AI algorithms to predict the likelihood of therapeutic response—are widely adaptable to other kidney conditions. AI-based systems could similarly inform decisions about drug selection, dose adjustments, and timing of interventions and move practice toward genuinely personalized medicine for pediatric kidney diseases.

## Personalized therapy

More sophisticated and powerful computational models and “omics” research promising more precise patient-level interventions have spurred and intensified efforts to personalize pharmacotherapy and dialysis regimens in nephrology over the past decade. Nanga et al. [27] illustrate this with a meta-analysis–based population pharmacokinetic (PK) model to guide tacrolimus dosing for solid organ transplant recipients. By applying a systematic “top-down” approach to patient-level data across multiple transplanted organs and clinical conditions with non-linear mixed-effects modeling (NONMEM) software, the authors identified key covariates (e.g., transplanted organ type, time post-transplant, and body weight) that significantly influenced tacrolimus clearance and distribution. Their internal and external validations—spanning adult and pediatric cohorts—demonstrated the tool’s potential for guiding PK-based tacrolimus dosing with the goal of reducing drug toxicity while ensuring adequate immunosuppression. Likewise, Zaza et al. applied “omics” methodologies to refine immunosuppressive regimens to prolong allograft survival in kidney transplant recipients [28]. While recent immunosuppressant protocols have improved short-term outcomes, long-term graft function remains suboptimal due to chronic drug-related toxicities and other factors. The authors propose that comprehensive genomic, proteomic, and metabolomic profiling could elucidate individual susceptibility to adverse events, guiding tailored immunosuppressive therapy that balances efficacy with toxicity mitigation. AI offers a powerful method for addressing the complex challenges in processing, analyzing, and interpreting omics data, as well as integrating multi-omics with clinical data [28]. However, they caution that further research and technological refinements are needed to seamlessly integrate multi-omic data into routine clinical workflows.

Finally, personalization extends to dialysis prescriptions, as evidenced by Galli et al. [29] and Niel et al. [30]. Galli et al. used specialized modeling software (Patient on-Line) to optimize automated peritoneal dialysis (APD) parameters—such as dwell times and glucose concentrations—based on each patient’s peritoneal membrane transport classification and residual diuresis. By individualizing the dialysis program, they achieved significant gains in weekly peritoneal Kt/V and creatinine clearance without increasing treatment burden or compromising residual kidney function. Niel et al. developed a neural network that utilized bio-impedance, blood volume monitoring, and blood pressure measurements as inputs to generate an artificial intelligence-derived dry weight output, which proved to be more accurate than the nephrologist’s estimation and resulted in improved blood pressure management in pediatric hemodialysis patients.

These studies highlight the broader trend of employing predictive algorithms and data-driven models to fine-tune therapies—whether medication dosing or dialysis regimens—and underscore the promise of fully personalized nephrology care.

## Disease prediction

There is significant untapped potential for AI-driven predictive treatment models in pediatric nephrology, even though their success in other medical domains has already been demonstrated. In fields such as oncology and cardiology, AI tools are being used to analyze large volumes of patient data—ranging from genomic information to longitudinal clinical records—to forecast individual responses to different therapeutics and optimize dosing regimens. These AI platforms often outperform traditional approaches by rapidly recognizing patterns and subtle risk factors that might otherwise go undetected. Pediatric nephrology, however, has been slower to adopt these technologies, in part due to the relatively smaller patient populations. Smaller patient populations create multiple challenges that collectively contribute to the slower pace of innovation. First, AI models typically require large, diverse datasets to capture the full spectrum of disease presentations and variability; however, smaller cohorts can limit both the quantity and quality of data, making it difficult to develop and validate robust models. Furthermore, small sample sizes carry a higher risk of overfitting and reduce statistical power, potentially compromising the generalizability of findings across diverse pediatric subpopulations. In addition, specialized pediatric nephrology centers are fewer in number, often widely dispersed, and may lack the dedicated informatics or computational resources needed to build and implement AI tools. When multi-center collaborations are pursued to overcome these limitations, they involve further logistical complexities, such as data standardization and strict governance protocols. Finally, heightened ethical and regulatory considerations—especially around consent processes for minors—can slow data collection and model development. Harnessing predictive AI algorithms in this specialty could provide a much-needed opportunity to personalize care for children with kidney disease by proactively identifying which treatments have the highest likelihood of success and least risk of adverse effects. Bridging this gap will require robust data-sharing frameworks, multidisciplinary collaboration, and dedicated validation studies to ensure that AI solutions are both accurate and generalizable in the pediatric nephrology setting [31, 32].

AI has great potential use in the prediction of diseases and outcomes [33]. An increasing number of studies focus on using different AI methods to predict AKI [34–36]. In pediatrics, Dong et al. [37] developed a ML model, trained on EHR data, to predict AKI before the traditional KDIGO creatinine-based criterion is met. The model successfully predicted 58% of patients who go on to develop AKI Stage 2/3 with a median lead time of 30 h. Similarly, Fragasso et al. [38] used AI to detect AKI that would occur within 48 h of pediatric cardiac surgery. The AI system had a positive predictive value of 94% and a negative predictive value of 97% for severe AKI in a population with a 43% prevalence of severe AKI. Fragasso does not report the lead time, but the methods suggest at least a 6-h pre-AKI detection. Whether these lead times in early AKI detection provide enough time to administer preventive or mitigating interventions is uncertain. The study discusses how AI was used to monitor pediatric cardiac surgery patients for AKI. The AI system assessed various risk factors and patient data in real time to alert healthcare providers about the potential development of AKI significantly before traditional diagnostic criteria would have identified the condition.

CKD in children is a particularly challenging condition to manage due to its subtle onset and progression. AI shows substantial promise in enhancing CKD management, particularly in addressing complex conditions like CKD–mineral bone disorder (CKD-MBD) and optimizing patient care through personalized strategies. For instance, quantitative systems pharmacology (QSP) models integrated with reinforcement learning (RL) have demonstrated effectiveness in simultaneously controlling serum calcium, phosphorus, and parathyroid hormone (PTH) levels, thereby achieving therapeutic goals more efficiently than conventional approaches [39]. Moreover, AI-based nutritional care platforms, such as the “Internet + hospital-to-home (H2H)” model, leverage improved wavelet transform algorithms for medical imaging and have been shown to significantly enhance nutritional status, biochemical markers, and overall quality of life in patients with advanced CKD [40]. In parallel, ML methods of predicting CKD progression and the need for kidney replacement therapy (KRT) have attained high accuracy in time-to-event models, offering valuable insights into disease trajectories [41, 42]. Additionally, mobile app-based intelligent care systems that employ deep learning and optical character recognition (OCR) can further reduce adverse outcomes by providing personalized, AI-guided interventions directly to patients’ smartphones, facilitating real-time decision support and remote monitoring [43]. Altogether, these examples illustrate how AI-driven modeling, ML-based predictions, and interactive digital health solutions can revolutionize CKD management—enabling more precise, timely, and patient-centered care. Clinicians are employing AI systems to monitor disease progression and predict future complications, including CKD stage V. These systems analyze longitudinal patient data and detect patterns that might indicate rapid progression and necessitate changes in treatment plans. For example, AI techniques show promise in analyzing imaging data and clinical data to predict CKD progression [42, 44–46].

Additionally, AI has shown tremendous promise in enhancing kidney transplant outcomes and decision-making through improved prognostic accuracy, risk stratification, and personalized care strategies. For instance, Ali et al. developed advanced AI-based models that outperform traditional indices (e.g., Kidney Donor Risk Index and Living Kidney Donor Profile Index) by employing ML and deep Cox mixture models to predict graft survival for both deceased- and live-donor transplants, thus enabling better donor-recipient matching and selection [47–49]. Furthermore, recent work on delayed-graft function (DGF) risk prediction has highlighted how AI-driven classifiers can efficiently incorporate key donor and recipient variables to improve DGF prognostication and subsequent care management [50]. In the realm of kidney transplant pathology, Bülow et al. illustrated how AI can automate lesion quantification in digital nephropathology, potentially standardizing diagnoses and tailoring post-transplant treatment plans [51]. Finally, Raynaud et al. demonstrated a dynamic prediction system that continuously updates kidney transplant recipients’ survival probabilities using repeated and real-time measurements of estimated glomerular filtration rate (eGFR) and proteinuria [52]. Collectively, these studies underscore the power of AI-driven approaches in guiding clinical decisions and optimizing long-term graft survival in kidney transplantation.

## Enhancing research in pediatric nephrology

AI technologies have proven to be transformative across many facets of nephrology, and their potential to bolster both patient care and research efforts in pediatric kidney disease is increasingly clear. By automating time-consuming administrative tasks, AI tools allow physicians more bandwidth to focus on the nuanced needs of young patients, fostering earlier detection and intervention for diseases. In parallel, predictive models that incorporate rich clinical, imaging, and genetic data not only enhance diagnostic accuracy but also guide more personalized, adaptive treatment strategies, thereby minimizing adverse outcomes. The real-time insights provided by ML and DL algorithms empower nephrologists to optimize medication dosing, predict disease progression, and promptly tailor therapies to each child’s unique risk profile. Furthermore, AI-based platforms can accelerate discoveries by sifting through extensive datasets to identify biomarkers, uncover novel disease pathways, and suggest innovative therapeutic targets. As these tools increasingly integrate multi-omic data—ranging from genomics to proteomics—they pave the way for precision medicine initiatives that elevate pediatric nephrology to a new standard of care. Beyond the clinic, AI-driven research accelerates hypothesis testing in silico, significantly decreasing the time and costs typically associated with pre-clinical and clinical trials. This synergy between patient care and scientific exploration underscores the transformative power of AI in improving outcomes for children with kidney diseases, sparking hope that ongoing advancements will revolutionize the field in the years to come.

AI is also playing a pivotal role in advancing the pace of pediatric nephrology research discovery. It is instrumental in sifting through extensive datasets to uncover patterns and insights that might take humans much longer to identify, if at all. AI tools help researchers identify potential biomarkers for disease, predict disease trajectories, and even generate hypotheses for new therapeutic approaches. The use of AI in clinical trials can refine recruitment processes by identifying candidates who match precise inclusion criteria, thus potentially reducing the time and cost associated with clinical studies. Automating repetitive and routine tasks such as data entry, literature reviews, and preliminary data analysis can also allow researchers more time to focus on the more complex tasks associated with research. AI tools are particularly useful in genetic research, where they help in analyzing genetic information alongside clinical data to understand the genetic bases of kidney diseases. This integration can lead to the development of personalized medicine strategies where treatment plans are tailored to the individual genetic makeup of a patient. By correlating genetic markers with disease phenotypes, AI models facilitate targeted therapies that are more effective and have fewer side effects. AI is also revolutionizing drug development and repurposing by predicting the efficacy and safety of potential drug treatments. AI algorithms can simulate drug interactions at the molecular level, speeding up the drug discovery process and reducing the need for extensive clinical trials. The most widely publicized scientific breakthrough is the development of the Alphafold DM model using Google DeepMind that accurately predicts a protein’s 3D structure from its amino acid sequence and contains over 200 million entries freely available to the scientific community [53]. Additionally, AI can identify new uses for existing drugs, potentially offering new therapeutic options for treating kidney diseases in children [54].

## The road ahead: challenges and opportunities

AI is poised to play a transformative role in pediatric nephrology by enhancing diagnostic accuracy, personalizing treatment, and improving patient outcomes. Its ability to integrate and analyze diverse, large-scale datasets—from genetic and environmental information to patient-reported outcomes—can deliver more holistic insights into kidney diseases in children. Real-time data monitoring and predictive algorithms enable earlier interventions, tailored therapies, and more precise risk assessments, potentially shifting the focus from reactive treatment to proactive prevention.

Healthcare professionals must receive adequate training to use AI tools effectively, while ensuring that these technologies augment rather than replace the doctor-patient relationship. Data privacy, security, and algorithmic bias are critical concerns, particularly in the context of pediatric health data, necessitating robust validation, continuous monitoring, and the highest ethical standards to protect the safety and well-being of young patients.

Moreover, regulatory frameworks must evolve to accommodate AI’s rapid advancements, while clinical workflows should be adapted to maximize AI’s benefits and minimize disruptions to care delivery. Despite these challenges, AI holds immense promise for reducing clinician burnout, optimizing research, and streamlining administrative tasks, ultimately freeing specialists to dedicate more time to direct patient care. With thoughtful implementation and ongoing oversight, AI can significantly bolster the capabilities of pediatric nephrologists, fostering a future of more precise, efficient, and compassionate care for children with kidney diseases.

By embracing AI responsibly, the pediatric nephrology community can harness its full potential to revolutionize healthcare, improving outcomes and ensuring that care remains grounded in human empathy. This forward-thinking approach will not only support more accurate and timely diagnoses but also facilitate personalized treatment plans, leading to better long-term patient experiences. Pediatric nephrologists will continue to play a vital role in guiding patient care, even as AI becomes increasingly integral to clinical practice. Rather than supplanting clinicians, AI tools serve as powerful adjuncts to clinical expertise, enhancing decision-making while leaving the nuanced interpretation of patient context and values firmly in the hands of the physician. Far from being mere “data editors,” pediatric nephrologists will integrate AI-driven insights with their unique understanding of each child’s physiology, family circumstances, and long-term treatment goals. This human-centered approach ensures a balance between technology’s potential for efficiency and accuracy, and the empathetic, individualized care that is essential in pediatrics. Progress in integrating AI into pediatric nephrology remains slower than in some other fields, due in part to smaller patient populations, data scarcity, and heightened ethical and regulatory complexities. Despite these challenges, AI’s value is maximized when clinicians, supported by adequate training and ethical guidance, employ algorithmic insights within a compassionate and holistic care framework. As the field continues to advance, AI stands ready to offer new, data-driven insights that can shape a more patient-centric, innovative, and effective standard of care.

## Supplementary information

Below is the link to the electronic supplementary material.

Graphical abstract (PPTX 2.09 MB)
