# Supplementary material for: AI-powered insights in pediatric nephrology: current applications and future opportunities
Source: Pediatr Nephrol. 2025 Sep 16;41(5):1275–86. doi: 10.1007/s00467-025-06911-1 (PMC13009068; doi:10.1007/s00467-025-06911-1)
Supplement: Supplementary file 1 — Graphical abstract (PPTX 2.09 MB) [file 467_2025_6911_MOESM1_ESM.pptx]

## Slide 1
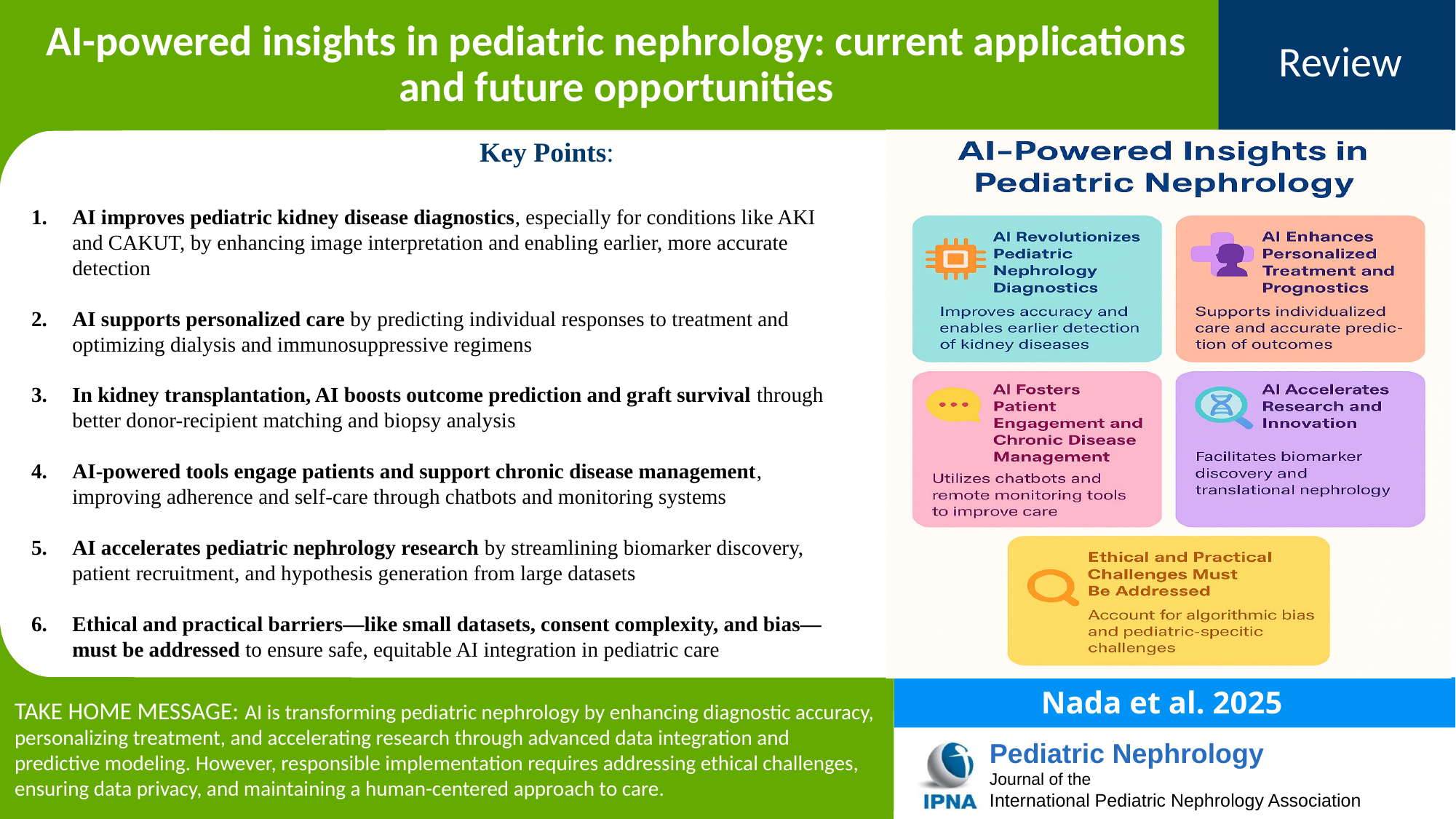

AI-powered insights in pediatric nephrology: current applications and future opportunities
Key Points:
AI improves pediatric kidney disease diagnostics, especially for conditions like AKI and CAKUT, by enhancing image interpretation and enabling earlier, more accurate detection
AI supports personalized care by predicting individual responses to treatment and optimizing dialysis and immunosuppressive regimens
In kidney transplantation, AI boosts outcome prediction and graft survival through better donor-recipient matching and biopsy analysis
AI-powered tools engage patients and support chronic disease management, improving adherence and self-care through chatbots and monitoring systems
AI accelerates pediatric nephrology research by streamlining biomarker discovery, patient recruitment, and hypothesis generation from large datasets
Ethical and practical barriers—like small datasets, consent complexity, and bias—must be addressed to ensure safe, equitable AI integration in pediatric care
Nada et al. 2025
TAKE HOME MESSAGE: AI is transforming pediatric nephrology by enhancing diagnostic accuracy, personalizing treatment, and accelerating research through advanced data integration and predictive modeling. However, responsible implementation requires addressing ethical challenges, ensuring data privacy, and maintaining a human-centered approach to care.
